# An Updated Review of the Anticancer Mechanisms and Therapeutic Potential of Naringenin

**DOI:** 10.1002/fsn3.70626

**Published:** 2025-07-15

**Authors:** Ahmad Mujtaba Noman, Muhammad Tauseef Sultan, Hassan Raza, Muhammad Imran, Muzzamal Hussain, Ahmed Mujtaba, Ehab M. Mostafa, Mohammed M. Ghoneim, Samy Selim, Soad K. Al Jaouni, Mohamed A. Abdelgawad, Tadesse Fenta Yehuala, Suliman A. Alsagaby, Waleed Al Abdulmonem

**Affiliations:** ^1^ Department of Human Nutrition, Faculty of Food Science and Nutrition Bahauddin Zakariya University Multan Pakistan; ^2^ TIMES Institute Multan Multan Pakistan; ^3^ Department of Food Science and Technology University of Narowal Narowal Pakistan; ^4^ Department of Food Sciences Government College University Faisalabad Faisalabad Pakistan; ^5^ Department of Food Science and Technology, Faculty of Engineering Sciences and Technology Hamdard University Islamabad Campus Islamabad Pakistan; ^6^ Department of Pharmacognosy, College of Pharmacy Jouf University Sakaka Saudi Arabia; ^7^ Pharmacognosy and Medicinal Plants Department, Faculty of Pharmacy (Boys) Al‐Azhar University Cairo Egypt; ^8^ Department of Pharmacy Practice, College of Pharmacy AlMaarefa University Ad Diriyah Riyadh Saudi Arabia; ^9^ Department of Clinical Laboratory Sciences, College of Applied Medical Sciences Jouf University Sakaka Saudi Arabia; ^10^ Department of Hematology/Oncology, Chair of Prophetic Medicine Application, Faculty of Medicine King Abdulaziz University Jeddah Saudi Arabia; ^11^ Department of Pharmaceutical Chemistry, College of Pharmacy Jouf University Sakaka Saudi Arabia; ^12^ Faculty of Chemical and Food Engineering, Bahir Dar Institute of Technology Bahir Dar University Bahir Dar City Ethiopia; ^13^ Department of Medical Laboratory Sciences, College of Applied Medical Sciences Majmaah University AL‐Majmaah Saudi Arabia; ^14^ Department of Pathology, College of Medicine Qassim University Buraidah Kingdom of Saudi Arabia

**Keywords:** anticancer, antioxidant, flavonoid, IL‐6, Naringenin, NF‐κB

## Abstract

The revolution in the 21st century has witnessed the development of the industrial and healthcare systems. Human health and well‐being are still global challenges despite this modernization and advancement. Cancer is extensively affecting people, demanding special attention and an effective dietary approach. Bioactive compound‐based natural therapies are a significant strategy to reduce the cancer burden. Naringenin, a bioactive compound widely distributed in the citrus family and is known for several health‐promoting properties, including anticancer activity. The current review highlights the anticancer potential of Naringenin by exploring recent studies through various databases like Google Scholar, PubMed, ScienceDirect, and Web of Science. The analyses revealed that Naringenin can inhibit cell proliferation and oncogenesis through different molecular mechanisms and signaling pathways like apoptosis induction, cell cycle arrest, and modulation of P13K/AKT/mTOR, TGF‐β1/Smad3, NF‐κB, TLR4, and MAPK pathways. Moreover, it can inhibit inflammatory markers (IL‐6, TNF‐α, and IL‐1β), downregulate oncogenes (Ras, HER2, MYC, and BCR/ABL1), and upregulate tumor suppressor genes (TSGs) such as TP53, PTEN, and BRCA1 and BRCA2 expression. Combining Naringenin with other bioactive compounds, chemotherapy drugs, and nanoformulations is a novel way to enhance its anticancer activity. However, poor bioavailability, stability, and lack of clinical trials and in silico studies hinder its therapeutic potential. Therefore, advanced techniques to enhance its stability and clinical trials regarding anticancer potential are required to validate its promising efficacy. The current review focuses on Naringenin's bioavailability, antioxidant, and anticancer potential through possible mechanisms.

## Graphical abstract

Naringenin is a bioactive compound widely distributed in the citrus family, belonging to polyphenols known for several health‐promoting properties, including anticancer activity. Several evidence‐based reports have proved that Naringenin can reduce tumorigenesis via inhibiting NRF2, TNF, NF‐κB, NR3C1, P13K/AKT, TGF‐β1/Smad3, and Bcl‐2 expression. Moreover, Naringenin's anticancer potential is due to its ability to regulate growth factors like IGF, EGF, FGF, and tumor suppressor genes (TSGs) such as p53, PTEN, and Caspase‐9 and Caspase‐3 enzymes activity. The current review focuses on the bioavailability, antioxidant, and anticancer potential of Naringenin through possible mechanisms.

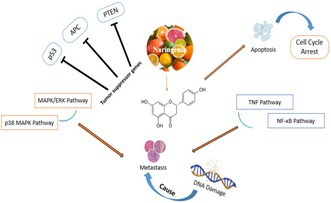

## 1 Introduction

Cancer is a rampant global challenge affecting millions of people worldwide, and in the last few decades, this burden has rapidly increased. Cancer is commonly referred to as uncontrolled and abnormal cell growth, associated with several risk factors such as genetics, sedentary lifestyle, dietary choices, pathogens, toxins, radiations, drugs, and chemicals, leading to morbidities and mortalities. Multiple cancers, that is, lung cancer (LC), colorectal cancer (CRC), gastrointestinal cancer, breast cancer, liver cancer, and kidney cancer, are significantly contributing to prevalence (Elsori et al. 2024). According to the International Agency for Research on Cancer (IARC), 20 million cancer cases were reported, and 9.7 million died from cancer in 2022, and lung carcinoma is leading them all. The estimations propose that around one in 5 men/women develop cancer in their lifetime, whereas almost one in 9 men and one in 12 women die from it (Bray et al. 2024). The pathophysiology covers complex mechanisms; however, oxidative stress and inflammation are fundamental contributors to cancer progression. Inflammation is the body's biological response to pathogenic infections, injuries, and chemical exposure to re‐establish homeostasis and avert tissue function loss (Hossain and Kubes 2019). The immune cells respond to this insult and try to recover the inflammation; however, sometimes impaired immunity fails to do so, leading to chronic inflammation. Studies have confirmed that this chronic inflammation is the key contributor to cancer development via damaging DNA, genetic modifications in oncogenes (rasH, rasK, and rasN), dysregulating inflammatory pathways (NF‐κB, MAPK, and JAK–STAT), and promoting pro‐inflammatory mediators (IL‐6, IL‐1β, IL‐8, IL‐17, and TNF‐α) (Chakraborty et al. 2020).

Diet therapy to ameliorate oxidative stress and inflammation through scavenging free radical species is an appropriate, affordable, and available source. The diet, including fruits, vegetables, herbs, and cereals with a natural background, comprises numerous bioactive compounds, which act as antioxidants and have anti‐inflammatory potential. Therefore, the intake of these natural products can remarkably reduce the cancer burden (Matsushita et al. 2020). Flavonoids are plant‐derived polyphenolic compounds known for their antioxidant, anti‐inflammatory, and anticancer properties. They modulate cellular signaling pathways, improve immune response, and protect against oxidative stress. Because of these properties, flavonoids show promising therapeutic potential in treating chronic diseases like cancer and other metabolic disorders (Rana and Mumtaz 2025). Naringenin (2S)‐4′,5,7‐Trihydroxyflavan‐4‐one, a hydrophobic flavonoid with the formula (C15 H12 O5), discovered in 1907 by Power and Tutin, primarily present in citrus, especially in grapefruit, lemon, and oranges, is soluble in organic solvents, having a molecular weight of 272.25 g/mol (Bhia et al. 2021). Naringenin is derived from naringin or narirutin hydrolysis and is mainly present as aglycones. However, glycosylated and neohesperidoside forms are also present (Motallebi et al. 2022). Several previous studies have proved the therapeutic potential and pharmacological activities of Naringenin against various metabolic disorders such as antidiabetic (Li et al. 2019), hepatoprotective (Naeini et al. 2021), nephroprotective (Kahramanoğullari et al. 2024), cardio‐neuroprotective (Tayo et al. 2024), and anticancer (Sharma et al. 2025; Yıldırım et al. 2022).

The current review covers several aspects of Naringenin and cancer management. It focuses on Naringenin's anticancer potential through possible mechanisms like apoptosis, cell cycle arrest, and modulation of various signaling pathways. Moreover, the detailed in vitro and in vivo studies and Naringenin bioavailability are the highlights of this review. Lastly, this review's limitations and challenges, such as the bioavailability of compounds and strategies to enhance therapeutic activities and clinical trials, are also significant features.

## 2 Methodology

The methodology segment was designed to review the updated anticancer properties of Naringenin. Relevant peer‐reviewed articles were retrieved from databases and search engines such as Google Scholar, PubMed, ScienceDirect, Wiley Online Library, and Web of Science, along with keywords like Naringenin bioavailability, Naringenin and breast cancer, Naringenin and LC, Naringenin and CRC, and Naringenin and prostate cancer. Moreover, Boolean operators such as AND and OR were used to enhance the search. The current review covers the most recent studies (2015–2025); however, a few are old. Inclusion criteria: original research articles inspecting Naringenin's effects on cancer cells or murine cancer models, studies that explicated molecular mechanisms of Naringenin's action. Exclusion criteria: non‐English articles, studies using homeopathic substances, and research that provides for other compounds besides Naringenin. The findings were systematically analyzed to evaluate the therapeutic potential of Naringenin in cancer management.

### 2.1 Bioavailability of Naringenin

The bioavailability of bioactive compounds is crucial to attaining their maximum health benefits because numerous factors influence their bioavailability, from raw material to excretion from the body. The source and processing are primary aspects that can reduce bioavailability, followed by the consumer gastrointestinal environment. The pharmacokinetics and pharmacodynamics of Naringenin revealed that it is metabolized in intestinal cells or the liver in two phases by cytochrome P450 monooxygenases; phase I is oxidation/demethylation, and phase II is sulfation/methylation. Furthermore, it is documented that gut microbiomes can improve Naringenin and other flavonoid bioavailability by metabolizing them into phenolic and aromatic ring‐fission catabolites. The metabolized Naringenin is mainly detected in urine and feces because urine is the basic and primary route of excretion (Kay et al. 2017). Regarding this, Zeng, Su, et al. (2019) used a single dose of 42 mg/kg D4‐naringin in rats and found 21 flavonoid metabolites and 11 phenolic catabolites in the urine and feces of rats. They reported four types of metabolites (D4‐Naringenin, D4‐hippuric acid (D4‐HA), D4‐3‐(4′‐hydroxyphenyl) propionic acid (D4‐HPPA), and D4‐p‐coumaric acid (D4‐p‐CA)) in urine, whilst only one (D4‐HPPA) in feces.

The studies have verified that the pure form of Naringenin follows a similar fate as Naringenin derived from Naringin. In the small intestine, phase II enzymes metabolize Naringenin to produce flavanone glucuronides and sulfates, but Naringenin metabolism in the stomach and colon is subtle (Najmanová et al. 2020). Orrego‐Lagarón and colleagues investigated the Naringenin metabolic profile in the stomach and colon of mice by liquid chromatography/electrospray. They reported that colonic samples contain more metabolites, such as Naringenin glucuronides and Naringenin sulfate, than gastric samples. This is due to greater glucuronosyltransferase and sulfotransferase activity in the colon or participation from colonic microbiota; however, apigenin was found in both samples (Orrego‐Lagarón et al. 2016). Besides GIT, the unaffected Naringenin can be present in the trachea, plasma, lung, heart, kidney, muscle, spleen, and brain within 24 h. After absorption and metabolism in GIT, metabolites were distributed to the liver for additional metabolism. Different kinds of metabolites can be detected in various tissues, such as Naringenin glucuronides in plasma, free Naringenin, and Naringenin‐7‐O‐sulfate as main forms in GIT, liver, and some other tissues (Zeng et al. 2020).

Bioavailability and bioaccessibility of Naringenin from diverse food sources have been reported by Aschoff et al. (2016), as they conducted randomized cross‐trials and volunteers consumed orange juice or fresh oranges of the same batch, and juice encompassed 2.4‐fold more citrus flavanones. The 24‐h urine sample analysis proved the excretion of 1.7‐fold more Naringenin when consuming fresh oranges, proving more bioavailability of Naringenin from orange juice rather than whole fruit because of the fiber matrix of oranges. One of the most significant factors is age, which can adversely affect the bioavailability of Naringenin and even the pharmacokinetics (Cossart et al. 2021). The complications associated with age are delayed gastric emptying, altered peristalsis, variations in bowel surface area, declines in splanchnic blood flow, and modifications in cytochrome P450 activity. Zeng, Yao, et al. (2019) analyzed age's effect on Naringenin's absorption and availability in older rats (20 weeks) and young adult rats with a single dose of 42 mg/kg pure Naringenin. They concluded that the older rats excreted more Naringenin in urine and feces than the young ones. The distribution also varies according to age; aged rats have substantially higher levels of Naringenin in the lungs and trachea.

### 2.2 Antioxidant Potential

The antioxidant potential is the capability of a substance/compound to scavenge free radical species of oxygen and nitrogen. The reactive nitrogen and reactive oxygen species (RNS/ROS) are highly unstable and reactive, involved in damaging cell membranes, cellular organelles, and even DNA, thus leading to oxidative stress, inflammation, and metabolic disorders. The bioactive compounds scavenge these radicals and protect them from damage. The antioxidant potential of Naringenin was verified by Al‐Ghamdi et al. (2021), as they determined the antioxidant potential of Naringenin nanoparticles against cadmium (Cd) toxicity in Nile tilapia. The specimens were exposed to 5 ppm of cadmium chloride monohydrate for 3 weeks and treated with bulk Naringenin and Naringenin nanoparticles. They concluded a significant reduction of hepatic malondialdehyde (MDA) levels in both treatments, whereas the protective outcome of Naringenin nanoparticles was more prominent. Similarly, Xu et al. (2023) mentioned Naringenin antioxidant activity in diabetic cardiomyopathy. Oxidative stress could lead to chronic metabolic syndromes like diabetes; however, studies have proved the antioxidant and scavenging potential of Naringenin. Nguyen‐Ngo et al. (2019) examined Naringenin's antidiabetic, antioxidant, and anti‐inflammatory properties in an in vivo rat model and an in vitro human model. They reported Naringenin's antidiabetic and anti‐inflammatory role via reduced expression of inflammatory cytokines, improved antioxidant mRNA expression, and enhanced glucose tolerance.

Studies have proved that Nrf2 and NF‐κB signaling pathways are involved in cellular oxidative stress, and Naringenin can inhibit and downregulate the inflammatory pathways like ARG/RAGE, NOXs, and NF‐κB while increasing the activation of the Nrf2 signaling pathway (Rajappa et al. 2019). Additionally, Naringenin has also proved effective in reducing the expression of the NOX2 pathway. Previously, the study of Liu et al. (2016) demonstrated the antioxidant activity of Naringenin by inhibiting the TLR4 signaling pathway, COX2, TNF‐α, NOX2, IL‐6, and iNOS expression. Furthermore, it also downregulates NF‐κB and MAPK activation and increases ATF3 expression. Likewise, the study of Yang et al. (2021) verified the antioxidant role of Naringenin in cisplatin‐induced renal injury via reducing NOX4 protein, mRNA levels, apoptosis, and inflammation. Teng et al. (2018) stated the inhibitory role of Naringenin in AGEs' formation and age‐induced cellular oxidative stress in RAW267.4 cells. Wojnar et al. (2018) proved the antioxidative potential of Naringenin in type 1 diabetic rats through RAGE and NOX signaling pathways downregulation in cardiomyocytes.

### 2.3 In Vitro Studies

Multiple studies have shown the anticancer activity of Naringenin in various human cell line studies. The combined effect of Naringenin and cyclophosphamide was studied to elucidate breast cancer (MDA‐MB‐231) proliferation; it resulted in Naringenin‐induced apoptosis, BAX expression, and caspase 3 and 9 activities. However, cell viability, Bcl‐2, and IL‐6 expression were significantly ameliorated by the synergistic action of Naringenin and cyclophosphamide (Noori et al. 2020). Krishnakumar et al. (2011) investigated the cytotoxicity of Naringenin nanoparticles on human cervical cancer (Hela), and it was shown that 50 μg/mL Naringenin nanoparticles revealed relatively higher (14%) cell propagation than other concentrations (30 and 40 μg/mL). Fadholly et al. (2022) utilized WiDr cell (human colon cancer) lines to inquire about Naringenin tumor‐ameliorating potential by treating with different Naringenin concentrations (10, 20, 40, 60, 80 μg/mL). The IC50 value of 63 μg/mL indicated growth suppression of WiDr cells and induction of caspase‐3 expression. The anti‐cancerous effect of Naringenin on prostate cancer (PC3 and LNCaP) was explored by supplementing 5–50 μM Naringenin. The results depicted that Naringenin restricted cell growth by inducing apoptosis and reducing cell membrane potential (Salehi et al. 2019). The anti‐inflammatory and anti‐mutagenic activity of Naringenin liquid crystalline nanoparticles (500 μL) was evaluated against human airway epithelium (BCi‐NS1.1) and A549 cell lines. The results showed that Naringenin nanoparticles attenuated cell proliferation, viability, and migration in the A549 cell line (Wadhwa et al. 2021). Choi et al. (2020) explored the inhibitory potential of Naringenin on human melanoma cells (B16F10 and SK‐MEL‐28) by administering Naringenin (100, 200, and 400 μM) for 1 day. It was observed that melanoma cells' cell viability, proliferation, and growth were comparatively more restricted by 400 μM Naringenin than by other treatment concentrations. The anti‐proliferative mechanism of Naringenin was studied against hepatocellular carcinoma (HCC) by supplementing diverse concentrations (100, 150, and 200 μM) for 24 h. The results revealed that Naringenin (100 μM) remarkably suppressed cell viability and growth and induced mitochondrial‐mediated apoptosis in Hep G2 cells (Arul and Subramanian 2013). Table 1 shows the anticancer potential of Naringenin in various cancers via different pathways.

**TABLE 1:** Anticancer potential of naringenin in various cancers via different pathways.

Compound	Cancer type	Dose/concentration	Mechanism/pathway	Outcome	References
Naringenin	Breast cancer	0–500 μM	ERK, ROS	Inhibit cell growth and proliferation, apoptosis induction	Noori et al. (2020)
	Lung cancer	0300 μM	AKT/MMP	Tumor metastasis inhibition, apoptosis induction	Chang et al. (2017)
	Colorectal cancer	50 mg/kg	NF‐kB/p65, PI3K/AKT	Cell cycle arrest, lessen proliferation, apoptosis promotion	Dou et al. (2013)
	Prostate cancer	10–50 μM	ERK, (PI3K)/AKT	Cell cycle arrest in G1, apoptosis induction	Lin et al. (2014)
	Gastric cancer	(0, 5, 10, 20, 40, 80, and 160 μmol/L)	MMP, ROS, PI3K/AKT	Proliferation inhabitation and apoptosis promotion	Bao et al. (2016)

### 2.4 Anticancer Perspectives

**FIGURE 1:**
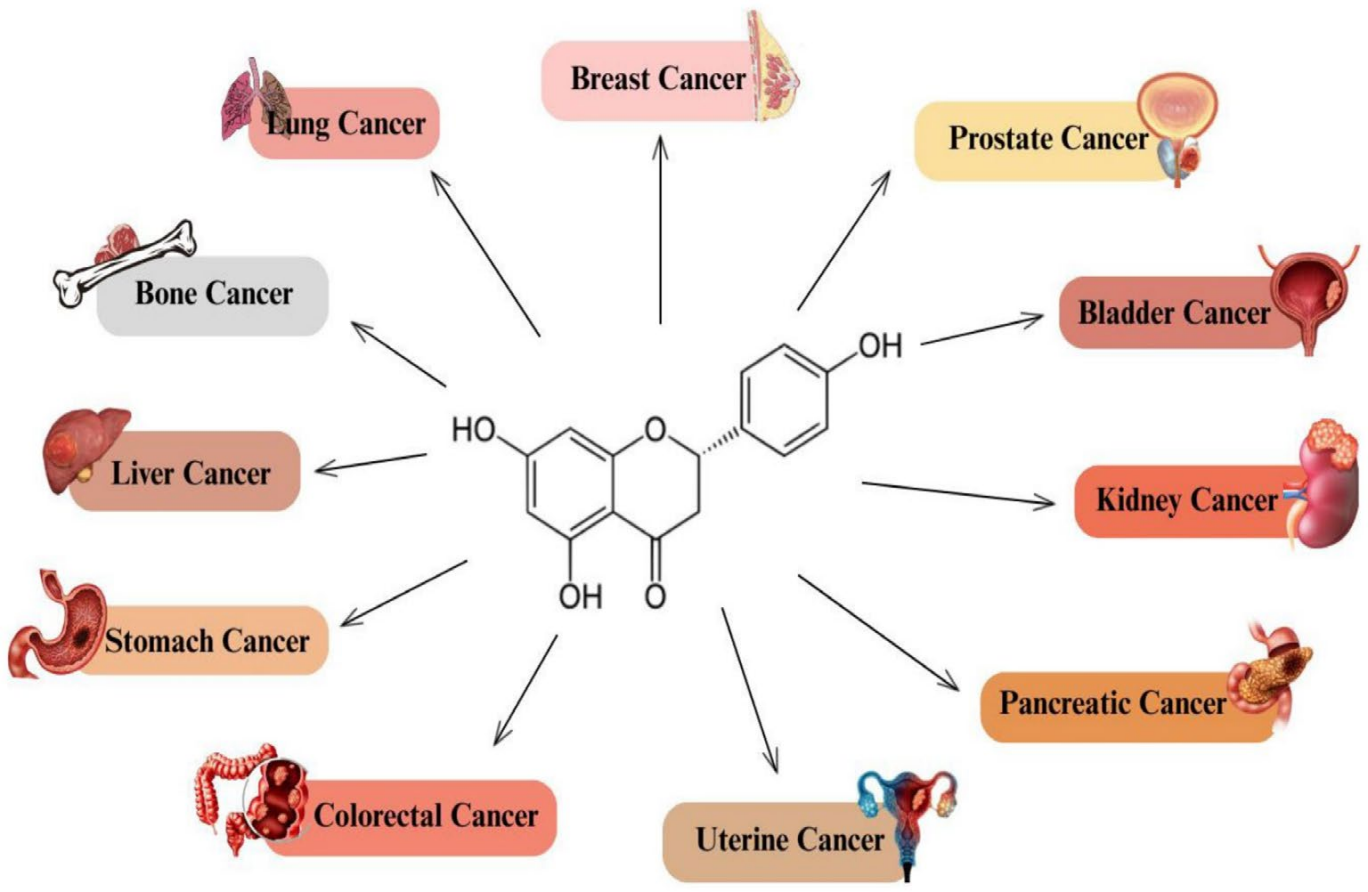
Anticancer activity of naringenin against various types of cancers.

**FIGURE 2:**
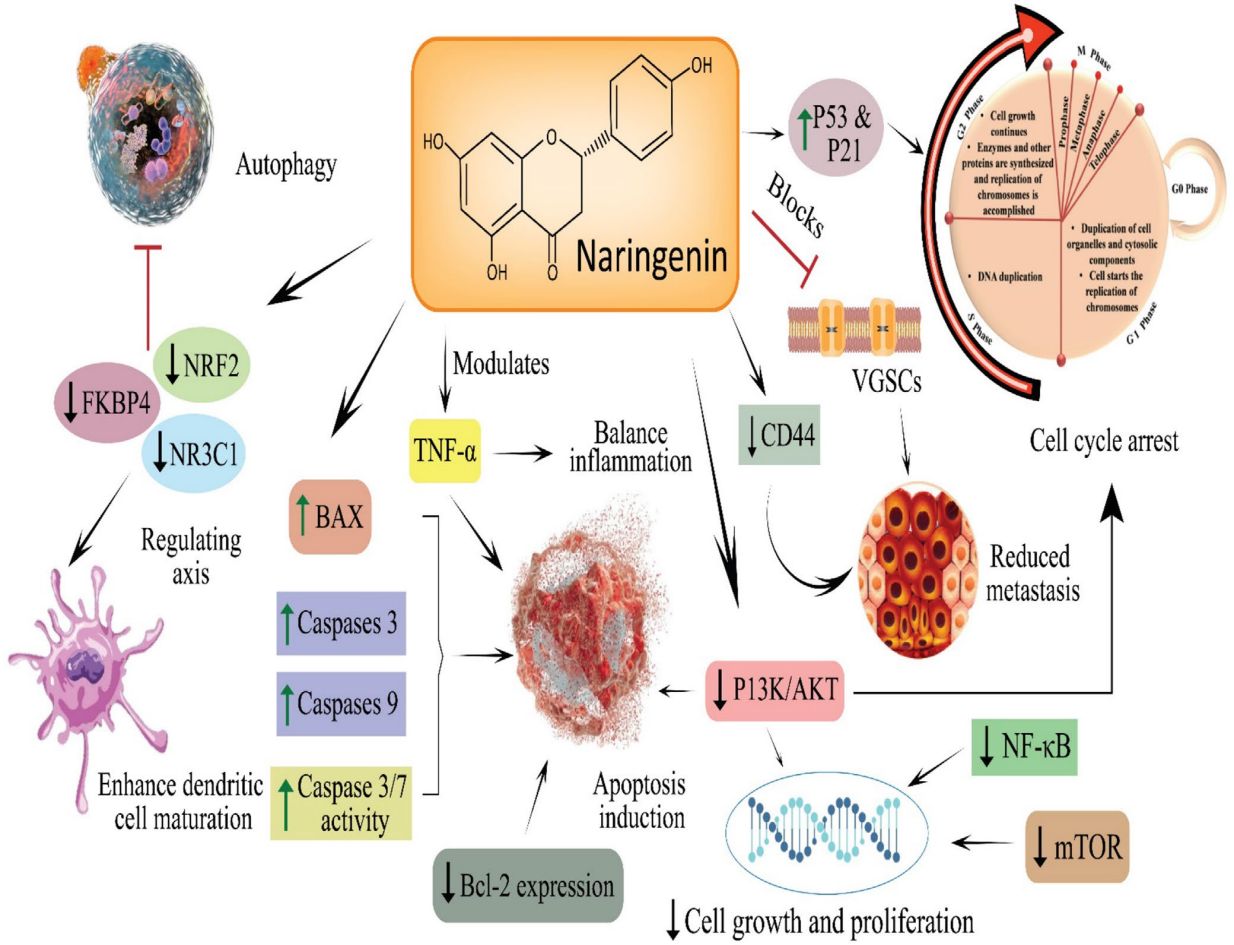
Anticancer activity of Naringenin: Upregulating the expression of anticancer mediators (BAX, caspase 3/7/9, P53, and P21), downregulating the expression of cancer development markers (Bcl‐2, PI3K/AKT/mTOR, NF‐κB, CD44, NRF2, and NR3Cl), apoptosis induction, and cell cycle arrest.

Cancer is one of the leading causes of fatalities globally. However, numbers are increasing in middle‐ and low‐income countries because of poor and unhygienic dietary choices, polluted environmental conditions, and prevailing pathogenic diseases. These factors progressively contribute to cancer progression through oxidative stress, inflammation, DNA damage, and genetic mutations. However, proper dietary strategies and integrative approaches can control and minimize the excessive burden. Secondary metabolites or phytochemicals from plant sources are natural compounds that can potentially decrease the risk factors, leading to cancer prevention. Naringenin, a polyphenolic component derivate of naringin, has been reported to have anticancer potential. The anticancer activity of Naringenin against various types of cancers is shown in Figure 1. Figure 2 shows Naringenin's potential to modulate molecular mechanisms and signaling pathways in cancer development.

### 2.5 Breast Cancer

Breast cancer (BC) is the 2nd leading cancer worldwide, and according to WHO reports, 2.3 million women are identified with BC, leading to 670,000 deaths in 2022. However, the numbers were low in 2018, with an estimated 2.089 million (Nardin et al. 2020). Several risk factors are documented for BC, such as gender, hormonal changes, age, physiological conditions, dietary patterns, and lifestyle behavior. Among these factors, hormonal changes are remarkably involved in cancer development. Estrogen is the fundamental hormone involved in the pathogenesis of cancer development. The high level and lengthy exposure to this hormone are linked with an augmented risk of cancer progression (Dall and Britt 2017). It has been documented that postmenopausal females with high estrogen levels are at greater risk of BC. Moreover, early menstruation (~12 years) and delayed termination (~50 years) increase the risk twofold compared to females who started late menstruation (~15 years) and completed it early (~40 years). Along with elevated estrogens, hormonal replacement therapy (HRT) is also a major risk factor. However, the risk can be reduced if HRT is used after 60 years (Cohain et al. 2017). The genetic risk factor accounts for only 5%–10% of cases of breast cancer development, and the known genes with mutations are BRCA1 and BRCA2 genes. These genes on chromosomes 17 and 13 are suppressor genes that sustain genomic stability, encrypt nuclear protein, and repair double DNA strand breaks by homologous recombination (Abdel‐Razeq et al. 2021). Mutations in other genes, i.e., CHEK2, ATM, PALB2, and BRIP1, display a moderate tendency to BC, and patients with these mutations have 2–3 times greater menace of developing malignant tumors (Chamseddine et al. 2022).

Drug resistance is a crucial problem in cancer therapy, leading to treatment failure and tumor recurrence. However, this obstacle can be overcome by targeting multiple pathways, a synergistic strategy, or by alternative natural treatment. Recently, Naringenin (100–200 μ M) alleviated tamoxifen resistance in MCF‐7 BC cells via impaired mitochondrial function and ROS‐mediated apoptosis. The findings showed that Naringenin augmented mitochondrial superoxide anions and hydrogen peroxide production while also causing mitochondrial dysfunction. Therefore, Naringenin could be a promising candidate to attenuate drug resistance in cancer cells (Eanes et al. 2025). Zhao et al. (2024) proved the anticancer potential of Naringenin, as they developed liver‐targeted Naringenin nanoparticles for BC endocrine therapy by improving estrogen metabolism in the liver. They reported that Naringenin (320 μM) suppressed HR+ breast cancer development up to 71.54% by triggering EST expression in the liver and declining estradiol levels in the liver, blood, and tumor tissue. Madureira et al. (2023) also mentioned Naringenin (30 mg/kg) and hesperidin's anticancer activity against BC, as they studied the antiproliferative activity of 99 flavonoids. The results revealed that both compounds reduced tumorigenesis through modulation of epigenetics, cell death induction via regulating apoptotic signaling pathways, and prohibition of tumor metastasis. However, the findings showed that Naringenin exhibited better anticancer potential than hesperidin. The studies on breast cancer tried to highlight the possible mechanisms and pathways that modulate and regulate further mediators involved in tumor progression. Considering this, NrF2 is a significant regulatory transcription factor involved in tumor immunity and carcinogenesis. Naringenin (50 mg/kg) effectively regulated the FKBP4/NR3C1/NRF2 axis to suppress the proliferation of BC cells in male BALB/c nude mice. Moreover, Naringenin could enhance dendritic cell (DC) maturation via the FKBP4/NR3C1/NRF2 axis (Xiong et al. 2022).

Rhman et al. (2022) evaluated the in vitro synergetic role of quercetin and Naringenin (0–250 μg/mL) against MCF‐7 breast cell lines. They used various concentrations of Que and Nar to assess cell viability. To evaluate oxidative stress and apoptosis, the IC50 was used to examine mitochondrial membrane potential, lipid peroxidation, Bcl‐2 gene expression, and caspase 3/7 activity. They concluded that IC50 (468 μg/mL) of Naringenin significantly produced cytotoxicity, decreased Bcl‐2 gene expression, and improved caspase 3/7 activity. Similarly, Askar et al. (2021) determined the in vitro and in vivo anticancer activity of Naringenin‐curcumin‐magnetic‐nanoparticles (CUR‐NAR‐D‐MNPs) via MTT assay. They reported that CUR‐NAR‐D‐MNPs (6.25–100 μg/mL) induce cell cycle arrest, apoptosis, subdued cell proliferation, and modulate TNF‐α, p53, P21, CD44, and ROS signaling. Female Sprague Dawley rats were administered with 1 mg/kg of CUR‐NAR‐D‐MNPs via gastric intubation, and it was found that CUR‐NAR‐D‐MNPs reduced tumor volume. Noori et al. (2020) claimed that Naringenin (0–500 μM) increased the anticancer potential of cyclophosphamide against the MDA‐MB‐231 BC cell line via upregulating BAX expression, activating caspases‐3 and caspases‐9, and declining Bcl‐2 expression. Wang et al. (2019) reported the anticancer activity of Naringenin (0, 10, 20, 40, and 60 μg/mL) in the MDA‐MB‐231 cancer cell line by reducing the viability of MDA‐MB‐231 cells by arresting the cell cycle at the G2 phase, apoptosis induction, and improving aspase3 and caspase‐9 activity. The anticancer potential of Naringenin in an in vivo/in vitro study of breast cancer is shown in Table 2.

**TABLE 2:** Anticancer potential of naringenin in in vivo/in vitro studies of breast cancer.

Cell lines/in vivo/in vitro	Dose/concentration	Mechanism/pathway	Results	References
In vivo	20 mg/kg	Triggering EST expression, declined estradiol levels in the liver	Inhibited HR+ breast cancer development and tumor tissue	Zhao et al. (2024)
In vivo	30 mg/kg	Apoptotic signaling pathways regulation, and prohibition of tumor metastasis.	Cell death induction, modulation of epigenetics	Madureira et al. (2023)
In vivo	50 mg/kg	FKBP4/NR3C1/NRF2 axis, maturation of dendritic cells	Suppress cell proliferation	Xiong et al. (2022)
MCF‐7	0–250 μg/mL	Decreased Bcl‐2 gene expression, improved caspase 3/7 activity	Produced cytotoxicity, lessened cell viability	Rhman et al. (2022)
MCF‐7, MTT assay	6.25–100 μg/mL	Modulate P53, P21, TNF‐α, CD44, and ROS signaling	Cell cycle arrest, apoptosis, subdued cell proliferation	Askar et al. (2021)
MDA‐MB‐231	0–500 μM	Upregulating BAX expression, activating caspases 3 and caspases 9 and reduced Bcl‐2 expression	Induced apoptosis	Noori et al. (2020)
MDA‐MB‐231	0, 10, 20, 40, and 60 μg/mL	Improved aspase 3 and 9 activities	Cell cycle arrest at G2 phase, apoptosis induction	Wang et al. (2019)

### 2.6 Pancreatic Cancer

The pancreas is a vital organ in the body performing dual functions as exocrine and endocrine glands. As an endocrine gland, it releases the insulin hormone, which controls and regulates blood glucose levels. The insulin and other endocrine hormones such as glucagon, somatostatin, proinsulin, pancreatic polypeptide (PP), amylin, and C‐peptide are produced from Langerhans islets (Karpińska and Czauderna 2022). Pancreatic carcinoma is the 4th leading cause of cancer mortality in the US. It may surpass CRC before 2040, mostly occurring from pancreatic duct cells and referred to as pancreatic ductal carcinoma (PDC) (Kanno et al. 2019). The etiology exposed that smoking, age, diabetes, obesity, *H. pylori* infection, chemical exposure, liver cirrhosis, and chronic pancreatitis are potential risk factors for carcinoma (Hu et al. 2021). It has been established that chronic pancreatitis (CP) is often associated with parenchymal fibrosis and irreversible lesions within exocrine and endocrine pancreatic tissue, ultimately leading to PDC. Chronic pancreatitis is involved in genetic mutation, and genes like PRSS1, FTR, SPINK1, TRPV6, and CTRC are once mutated and may cause PDC. Moreover, CP is linked with the modification of molecular mechanisms like TGF‐β signaling, Wnt/notch signaling, G1/S checkpoint, and KRAS signaling, and the modification of the KRAS oncogene on codon 12 is the starting event in several PDC cases, followed by the inactivation of INK4a, TP53, and DPC4 TSGs (Saiki et al. 2021; Buscail et al. 2020).

The Naringenin nanoparticles (0–60 μM) were evaluated via in vitro analysis to ameliorate PDC against the pancreatic cell line. The NPs showed IC50 (48.89, 44.70, and 32.08 μg/mL) to inhibit cell proliferation and alleviate oncogenesis (Akhter et al. 2020). Lee et al. (2019) studied the combined effect of Naringenin and hesperetin (50:50) against pancreatic cancer cell lines (Panc‐1, Detroit551, and SNU‐213) through FAK downregulation and the p38 signaling pathway. Furthermore, the combined strategy significantly reduced FAK phosphorylation and stopped human pancreatic cancer (HPC) cell migration. Moreover, BALB/c nude mice were supplemented with a (10 and 30 mg/kg) Naringenin–hesperetin mixture to reduce tumor size via caspase‐3 induction. Similarly, Park, Choi, et al. (2017) verified the anticancer effect of Naringenin against pancreatic cancer SNU‐213 cells through ASK1‐triggered apoptosis and ROS. They verified that Naringenin (200, 400, and 600 μM) suppressed the expression of peroxiredoxin‐1 and activated JNK, p38, ASK1, and p53 proteins in SNU‐213 cells. The transforming growth factor‐β (TGF‐β) signaling pathway is a critical modulator of Epithelial‐mesenchymal transition (EMT), which promotes cellular movement and metastasis during the embryonic stage and tumorigenesis. Moreover, numerous evidence verified that this process is Smad3‐dependent. Lou et al. (2012) stated that Naringenin (50 and 100 μM) downregulated EMT markers expression, i.e., vimentin, MMP2, N‐cadherin, and MMP9 in both mRNA and protein levels via hindering TGF‐β1/Smad3 signal pathway in aspic‐1 and panic‐1 pancreatic cancer cells.

### 2.7 Prostate Cancer

The prostate is a walnut‐shaped gland present below the bladder that creates and forces semen through the urethra when on ejaculation and gets larger with age. The common prostate gland problems are prostate cancer, prostatitis, and benign prostatic hyperplasia (BPH). Prostate cancer (PC) is the 2nd most prevalent cancer among men, and according to ACS, 299,010 new cases and 35,250 deaths from prostate cancer have been reported, which means 1 in 8 men can be diagnosed with prostate cancer during their lifetime (Gandaglia et al. 2021). Prostate cancer involves the transformation of various processes, including prostatic intraepithelial neoplasia (PIN), and along with this, androgenic regulation of prostate cancer is another fundamental process responsible for PC progression. Androgenic regulators (ARs) are critical transcription factors in cancer development through testosterone and DHT, thus leading to nuclear translocation of receptors, triggering transcription of genes, proliferation, cell differentiation, and apoptosis (Dahiya and Bagchi 2022). Various pathways, such as TGF‐β, IGF, EGF, and FGF, are dependent on ARs. The EGF, with its membrane‐related tyrosine receptor kinase EGF‐1, is responsible for the progression and intrusiveness of cancer cells by improved migration. Moreover, tumor suppressor genes like PTEN negatively regulate the PI3K‐AKT–mTOR pathway and inhibit the cell cycle at the G1 stage, hence hindering cell proliferation (Imada et al. 2021). Thus, the dysfunction of PTEN results in an upsurge in the PI3K‐AKT–mTOR pathway and impairs normal AR regulation, subsequent amplified proliferation, AR expression, and declined apoptosis.

Studies have proved that PIM‐1 kinase, which is involved in cellular development, immunoregulation, and oncogenesis, is an appropriate therapeutic target for prostate cancer. Serine/threonine (PIM‐1), a proto‐oncogene, is vital in cancer development and cell proliferation (Wang et al. 2017). Rathi et al. (2024) investigated the anticancer activity of Naringenin and quercetin (0, 10, 20, 30, 50, 75, 100, and 200 μM) via targeting of PIM‐1 kinase. They concluded that both bioactive components remarkably subdued prostate cancer cells (LNCaP) growth and proved potent PIM‐1 kinase inhibitors. The recorded IC50 for both compounds was 17.5 μM for Naringenin and 8.88 μM for quercetin. Thus, quercetin is more potent than Naringenin. Pearce et al. (2023) explored the anticancer potential of Naringenin gold nanoparticle extracts (200, 100, 50, and 12.5 μg/mL) against PC. They evaluated the effect on LNCaP and PC‐3 cell viability by MTT assay and reported a significant reduction in PC3 cells. Torricelli et al. (2021) assessed the combined treatment of α‐tocopherol (100 μM) and Naringenin (10 μM) in PC‐3 cell lines based on a combined strategy. They reported a significant reduction in oxidative stress, glyoxalase I (GI), GST, and GR activities and augmented TBARS level and SOD activity. Furthermore, combined treatment contributed to ornithine decarboxylase (ODC) expression reduction and the intracellular level of polyamines to mitigate oxidative stress. The PI3K/AKT pathway generally involves cell survival and proliferation and inhibits apoptosis. However, in certain conditions, such as the presence of external stimuli, it can act as pro‐apoptotic. Previously, the studies of Aktas and Akgun (2018) and Lim et al. (2017) proved Naringenin's (75 μM and 50 and 100 μM) anticancer role via blocking voltage‐gated sodium channels and PI3K/AKT and MAPK‐induced apoptosis, respectively.

### 2.8 CRC

According to WHO, CRC is the third leading cancer and ranks second in fatalities worldwide. The data for 2024 provided by ACS confirmed that 106,590 new cases of colon cancer and 46,220 new cases of rectal cancer were reported in the US. The development of tiny cell groups characterizes CRC, referred to as polyps inside the colon, and somehow, with age, these polyps convert into cancerous tumors in the next 5–10 years. The other risk factors associated with CRC are hereditary disorders, genetic mutations, inflammatory bowel illness, and other malignancies (Al‐Muswie et al. 2023). Recent advances in epigenetics discovered a missing link between some gene expression patterns related to CRC and the absence of genetic irregularities. In addition, microRNAs (miRNAs) influence cancer‐associated pathways at a post‐transcriptional level, and these miRNAs contribute to the beginning of CRC, progression, and metastasis. Along with epigenetics, the genetic alterations in oncogenes and TSGs cause dysplastic epithelium in the adenoma‐carcinoma process and result in CRC progression (Müller et al. 2016). Genetic and epigenetic modifications lead to amended CRC pathways, that is, CIN, MSI, and CIMP, which play critical roles in CRC development. Among these, CIN accounts for ~80%–85% of CRC cases and initiates growth‐promoting pathways, whereas decreased apoptotic pathways' activity (Fischer et al. 2019).

Besides these pathways, a series of other pathways are also involved in CRC, which are modulated and regulated by oncogenes and tumor suppressor genes. The TSG APC is generally changed in CRCs, and this mutation activates the Wingless/Wnt pathway. This Wnt signaling pathway further mutates KRAS and TP53, leading to the clonal development of polyp cells to cancer, followed by the TGF‐β1 mediated cell signaling pathway and accelerated CRC development (Novellasdemunt et al. 2015). More than half of CRC cases are due to altered KRAS and B‐Raf, which activate MAPK, WNT‐APC‐CTNNB1, PI3 K, TGFB1‐SMAD, and RAS–RAF–MAPK pathways and promote proliferation with inhibited apoptosis (Ahronian et al. 2015).

The estrogenic receptor beta (ERβ) prevents oncogenesis by triggering apoptosis. Thus, it can be a potential target to reduce tumor development and progression. Considering this, the study of Lozano‐Herrera et al. (2022) based on Naringenin fermented extract proved that Naringenin (IC50 250 μM) effectively improved Erβ, PTEN, and CASP9 expression, promoting apoptosis and modulating genes associated with the p53 signaling pathway, miR‐200c, and miR‐141 in the HT‐29 CRC cell line. Studies have proved that combined therapy, such as Naringenin with other bioactive compounds or chemotherapy drugs, has produced a more promising effect against cancer than single anticancer therapy. Regarding this, Zeya et al. (2022) explored the combined effect of Naringenin and diosmin (10–320 μM) in HCT116 and SW480 CRC cell lines by activating apoptosis and inflammatory pathways. The results showed that the combination significantly improved apoptotic pathway markers, that is, Bcl2, p53, caspase‐3, Bax, caspase‐8, and 9, and reduced inflammatory mediators like NFκβ, IKKα, and IKKβ. Moreover, both compounds synergistically induce cytotoxicity and cell cycle arrest in the G0/G1 phase. The CI values ranged from 0.96 to 0.76 for HCT116, and SW480 exhibited CI values ranging from 0.76 to 0.53. In addition, the decreased expression of mediators in HCT116 was 0.21, 0.068, and 0.169‐fold, whereas for SW480, the expression was 0.2, 0.11, and 0.14‐fold reduced compared with control. Song et al. (2016) proved that Naringenin has been associated with stimulated p38‐dependent ATF3 expression and apoptosis in both protein and mRNA levels in CRC. They treated HCT116, SW480, and HT‐29 CRC cell lines with 100 and 200 μM Naringenin and observed that the treatment reduced cell viability by 28% and 15% in HCT116 and SW480, respectively, whereas HT‐29 decreased by 45%. Therefore, Naringenin can be effective in attenuating CRC. Zhao et al. (2016) elaborated on the protective role of 6‐C‐(E‐phenylmethyl) Naringenin, a compound present in Naringenin‐fortified fried beef, against CRC. They concluded that 6‐CEPN reduced cell growth via cell cycle arrest in the G1 phase, triggering necrosis and autophagy in cancer cells. In addition, 6‐CEPN inhibited RAS activation and downregulated Icmt/RAS signaling pathways, thus exhibiting cytoprotective autophagy in cancer cells. Daniels et al. (2012) reported the anticancer activity of Naringenin via downregulation of MCT‐1, p21, and TLR‐4 expression in CRC cells.

**FIGURE 3:**
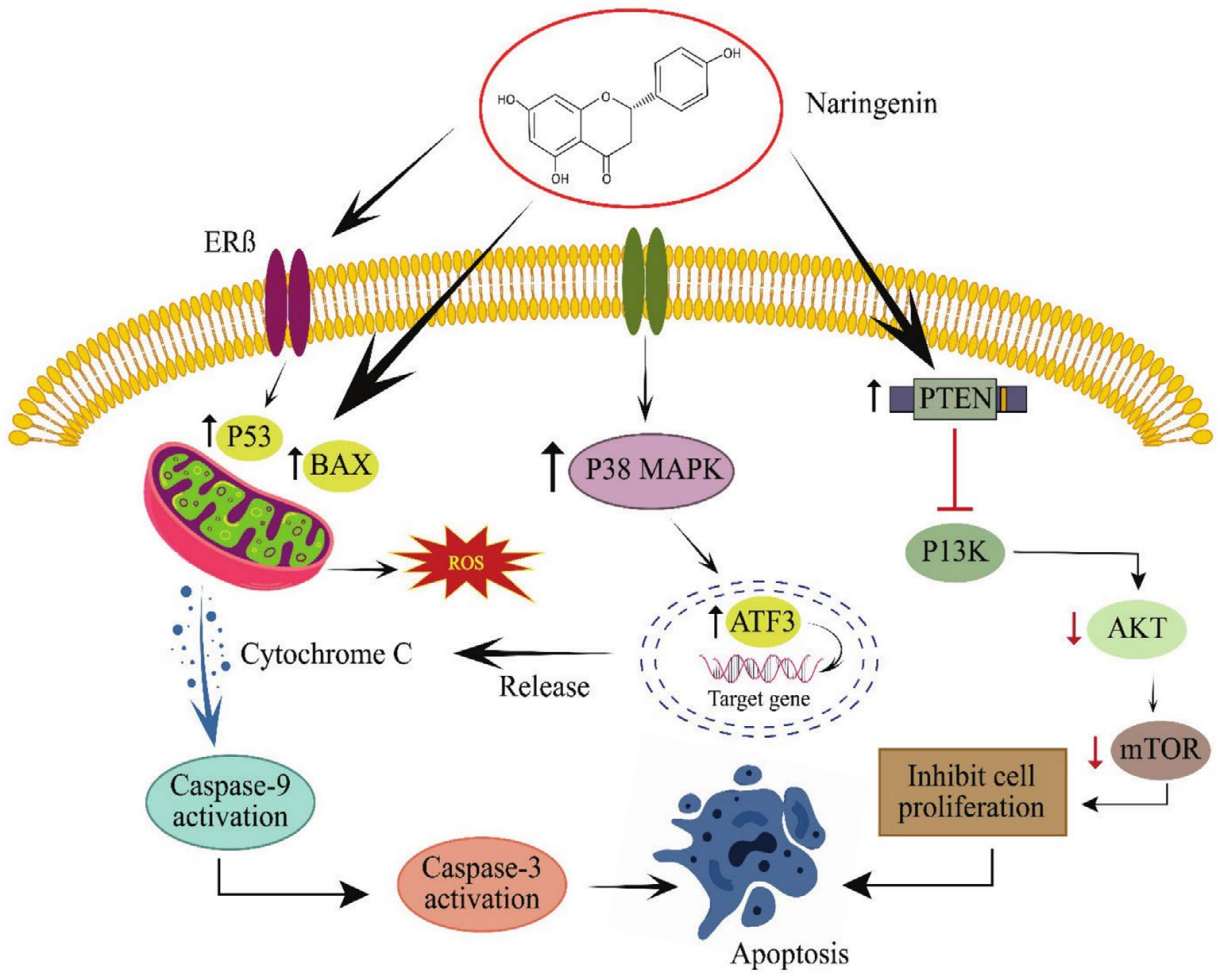
Anticancer activity of naringenin against CRC: Upregulating the expression of PTEN, p53, BAX, P38, ATF3, downregulating the AKT and mTOR pathways, and caspase 3/9 activation.

Recent studies have established that Naringenin can improve gut microbiota to ameliorate high‐fat diet‐induced CRC. Rodents (C57BL/6 male mice) were fed a high‐fat diet with 10 mg/kg i.p. azoxymethane once and then treated orally with Naringenin (100 mg/kg) for 10 weeks. The findings showed that Naringenin supplementation modulated the IL‐6/STAT3 pathway, improved claudin‐3, occludin, and ZO‐1 expression, reduced LPS, IL‐1β, IL‐6, and TNF‐α, and enhanced IL‐10 levels in mice (Sun et al. 2025). AMP‐activated protein kinase (AMPK) association with CRC is widely discussed, and it plays a crucial role in cell proliferation, apoptosis, and metastasis, thus making it a potential target for treating CRC. BALB/c‐nu mice were injected with LoVo cells and supplemented with Naringenin (10, 20, and 40 mg/kg) alone and in combination with 5‐fluorouracil (10, 20, and 40+ 12.5 mg/kg). Moreover, male BALB/c mice were injected with AOM/DSS to induce CRC and then treated with Naringenin (40 mg/kg) alone and combined with 5‐fluorouracil and compound c (40 + 12.5 + 20 mg/kg). The results revealed that combined therapy proved more effective than Naringenin alone. The combination induced apoptosis, inhibited cell proliferation, and activated AMPK phosphorylation. Moreover, the combined treatment increased ROS production, reduced mitochondrial membrane potential, and stimulated the AMPK/mTOR pathway (Wang, Zhou, et al. 2024). Anticancer activity of Naringenin against CRC: upregulating the expression of PTEN, p53, BAX, P38, and ATF3 and downregulating the AKT and mTOR pathways shown in Figure 3.

### 2.9 Liver Cancer

The liver is a vital organ in the abdominal cavity and plays a significant role in the metabolism and detoxification of xenobiotics. Besides, it produces several proteins, enzymes, and hormones. The physiological demonstration revealed that the lobule is the functional unit of the liver, which is hexagonal and composed of hepatocytes. The basic function of the liver is the production of bile, a fluid containing water, bile salts, electrolytes, bile acids, bilirubin, cholesterol, and phospholipids, which are involved in the digestion and absorption of lipids (Yang 2021). The cytochrome enzyme family is mainly involved in metabolism and detoxification mechanisms. The fat‐soluble vitamins enter the liver through intestinal absorption via chylomicrons and VLDL. In contrast, drugs and other xenobiotics transform into a hydrophilic form from lipophilic via two reactions. Phase I involves the formation of hydrophilic solutes via oxidation, reduction, and hydrolysis by the CYP450 family of enzymes. Phase II includes the conjugation of metabolites, formed in phase I, to convert them into hydrophilic compounds for secretion into blood and bile (Almazroo et al. 2017).

Liver cancer or hepatocellular carcinoma (HCC) is the sixth most common cancer globally, affecting 41,630 people (28,000 men and 13,630 women), and 29,840 people (19,120 men and 10,720 women) will die in 2024. Liver cirrhosis, fatty liver disease (FLD), alcohol consumption, exposure to toxins and chemicals, hepatitis, and congenital disorders are major risk factors related to HCC (Huang et al. 2021). The pathophysiology showed that different factors contribute differently to HCC pathogenesis. Viral hepatitis, a significant risk factor, mainly modifies genes such as TERT, PDGFR β, and MAPK1. Moreover, the virus alters other proteins like HBx, which can mutate the Ras, JNK, Raf, MAPK, and ERK gene expression (Wang, Yeh, and Chen 2021). Alcohol includes the production of pro‐inflammatory mediators via monocyte activation and results in augmented circulating endotoxin concentrations, triggering Küpffer cells, which release several chemokines and cytokines, including IL6, TNFα, IL1β, and prostaglandin E2 (Hillmer et al. 2020). The toxins, specifically aflatoxin, and other health hazard chemicals are also linked with gene mutation, especially TSG p53 expression. Besides p53 alterations, the other epigenetic modifications result in the dysregulation of TSGs and oncogenes, that is, β‐catenin, MET, ErbB, p16(INK4a), COX2, HGF, and E‐cadherin (Cao et al. 2022).

Studies have proved that Naringenin can ameliorate HCC through apoptosis and prevent oxidative damage. Li, Deng, et al. (2024) studied the anticancer activity of Naringenin (50 mg/kg) by inducing ferroptosis cell death through lipid peroxidation in BALB/c nude mice because Naringenin is involved in lipid metabolism modulation via AMPK. They found that Naringenin at non‐toxic doses (< 0.2 mM) substantially improves the anticancer activities of ferroptosis inducers like elastin, RSL3, and sorafenib in HepG2 and Hep3B cell lines. In addition, the reduction of aerobic glycolysis modulated by the AMPK‐PGC1α signaling axis is the main aspect of enhancing ferroptosis in liver carcinoma. Almoallim et al. (2024) evaluated the anticancer potential of ibuprofen/Naringenin nanoparticles in liver cancer. They concluded nanoparticles inhibit angiogenesis and proliferation with apoptosis induction in HepG2 cell lines. The CI of 0.623 and 0.155 for ibuprofen and Naringenin showed their significant anti‐tumor potential. Elnawasany et al. (2023) also investigated the anticancer effect of Boswellic acid, curcumin, and Naringenin‐loaded nanoparticles (NPs) against HepG2 cell lines and reported inhibition of HepG2 cell proliferation. The results showed that IC50 was 5.58 ± 0.27, 2.51 ± 0.11, and 5.21 ± 0.18 at 48 h for Boswellic acid, curcumin, and Naringenin NPs, respectively. Kang et al. (2019) explored the anticancer activity of Naringenin derivate (6‐CEPN) against NANOG+ cells in HCC via suppressing the Wnt/β‐catenin signaling pathway and upregulated GSK3β. The findings showed that IC50 (15–20 μM) of 6‐CEPN suppressed cell proliferation. Moreover, 6‐CEPN (25 mg/kg) body weight for 2 months reduced tumor growth and metastasis in nude BALB/c mice. Non‐alcoholic fatty liver disease (NAFLD) could be one of the major reasons to induce HCC. In a double‐blind, placebo‐controlled clinical trial, 100 mg of Naringenin (twice/daily) for 4 weeks was supplemented to 44 NAFLD patients. The study findings exhibited that Naringenin supplementation reduced oxidative stress and inflammation related to NAFLD via reducing liver lipid peroxidation and attenuated pro‐inflammatory markers expressions such as TNF‐α, IL‐6, and IL‐1β through inhibition of NF‐κB (Naeini et al. 2021).

### 2.10 LC

LC is the top reason for cancer mortality globally, comprising two main types: small cell LC (SCLC) and non‐small cell LC (NSCLC). According to ACS, the estimation of morbidities and mortalities in 2024 will be 234,580 new cases of LC and 125,070 deaths from LC. Smoking is a major risk factor for LC and is responsible for ~85% of cases. However, occupational chemical and heavy metal exposure, family history, and air pollution are also related to LC (Barta et al. 2019). The pathophysiology involves dysplasia of the lung epithelium due to smoking, and upon continuation, it may lead to genetic mutations and affect protein synthesis. The genetic mutation differs for both types, as a mutation in MYC, BCL2, and p53 genes for SCLC and p16, EGFR, and KRAS mutation for NSCLC (Lindeman et al. 2018). Other than this, the dysfunction of p107 and p130 (RB family), tumor suppressor PTEN, chromatin regulator CREBBP, and NOTCH receptors are significant factors that contribute to cancer development and pathogenesis (Lázaro et al. 2022). Chromosomal modifications are critical factors for DNA damage and gene mutation. The 3p14–23 chromosome deletion is often associated with SCLC (Ma et al. 2022). Moreover, initiating the PI3K–AKT–mTOR pathway has been associated with SCLC progression and apoptosis resistance (Kern et al. 2020).

Multiple studies have proved the anticancer activity of Naringenin concerning LC, so regarding this, Chang et al. (2024) studied the anticancer potential of Naringenin in H1299 and A459 cancer cells, and they concluded that Naringenin (250 μM) induced autophagy via modulating ROS generation, cell cycle arrest, recovered cyclin‐dependent kinase activity, reduced ratio of microtubule‐related proteins LC3II/LC3I, inhibited activity of the AMPK signaling pathway, and enhanced apoptosis. The human lung adenocarcinoma cell line (A549) was recently treated with 0–100 μM of Naringenin‐loaded iron oxide‐silica nanoparticles. The results showed that the 100 μM concentration of NPs proved cytotoxic to cells and caused 85% of cell apoptosis, whereas free Naringenin caused only 68% cell death. Moreover, the cancer cells exhibited cytoplasmic shrinkage and cellular structure alterations (Nagarajan et al. 2025). Amelimojarad et al. (2022) proved the anticancer and anti‐inflammatory role of combined Naringenin (50 μg/mL) and artemisinins (10–50 μg/mL) in the A549 cancer cell line via inhibiting cell development, reducing pro‐inflammatory cytokine levels, and augmenting PKA and cAMP levels. Recently, Wang, Zhang, et al. (2024) reported that Naringenin nanoparticles (NarNps) (IC50 271.0 μM) inhibited cyclin B1, alleviated cell proliferation, blocked cells at the G2/M phase, and suppressed the development of rheumatoid arthritis‐linked LC in A549 and H1299 cell lines. Moreover, 150 mg/kg Naringenin for 20 days in BALB/c nude mice significantly reduced tumor size, metastasis, and CCNB1 expression. In another study, Naringenin (100 μM) lowered and downregulated p21, pAkt, Akt, and MMP‐2 protein expression, reduced BCL2 and BCLXL mRNA levels, whereas increasing BAX mRNA expression, RAD50 protein levels, and CASPASE3 and caspase‐3 activity in NCL‐H23 LC cells (Baruah et al. 2020). Sun ZhenFeng et al. (2019) investigated Naringenin anticancer activity against the A549‐CSCs LC stem cell line. They stated that Naringenin (25, 50, and 100 μg/mL) significantly reduced the cell viability of A549‐CSCs, lowered the expression of Sox2 and Oct4 mRNA, and inhibited Notch1/Hes1 pathway expression. Table 3 shows the anticancer potential of Naringenin against various cell lines of lung carcinoma.

**TABLE 3:** Anticancer potential of naringenin against lung carcinoma.

Cell lines/in vivo/in vitro	Dose/concentration	Mechanism/pathway	Results	References
H1299, A459	25–500 μM	Inhibiting the AMPK signaling pathway, modulation of ROS generation	Induce autophagy, cell cycle arrest, apoptosis	Chang et al. (2024)
A549	50 μg/mL	Augmentation of PKA and cAMP levels	Inhibition of cell development, reducing pro‐inflammatory cytokines	Amelimojarad et al. (2022)
In vivo	—	Inhibition of cyclin B1, blocked cells at G2/M phase	Alleviated cell proliferation, suppressed development of rheumatoid arthritis‐linked lung cancer	Wang, Zhou, et al. (2024), Wang, Zhang, et al. (2024)
NCL‐H23	100 μM	Downregulated p21, pAkt, Akt, MMP‐2 protein expression, reduced BCL2 and BCLXL mRNA levels, increased BAX mRNA expression, RAD50 protein levels and CASPASE3 and caspase‐3 activity	Cell proliferation inhibition, apoptosis induction	Baruah et al. (2020)
A549‐CSCs	25, 50, and 100 μg/mL	Lowered expression of Sox2 and Oct4 mRNA, inhibited the Notch1/Hes1 pathway expression	Apoptosis, cell cycle arrest, reduced cell viability	Sun ZhenFeng et al. (2019)

### 2.11 Kidney Cancer

Kidneys are the main organs involved in the metabolism, filtration, absorption, and excretion of metabolites. Furthermore, various hormones and enzymes are produced by the kidneys. Despite its prime significance, the peril of renal cancer has been alarming the global population. According to ACS, kidney cancer is the 10th most common cancer in both genders, accounting for 4%–5%. It has been estimated that in 2024, 1610 new cases of kidney cancer will be diagnosed, and 14,390 people will die from this disease. Renal cell carcinoma (RCC) is a common type of renal cancer, and 9 out of 10 renal cancers are RCC. Several risk factors, including smoking, obesity/overweight, hypertension (HTN), congenital abnormalities, family history, chronic renal ailments, and exposure to certain chemicals, are responsible for cancer development (Scelo and Larose 2018). The pathogenesis of RCC is elaborated on as starting from the proximal renal tubular epithelium, and structural amendments occur on the short arm of the 3p chromosome. The genes linked with genetic mutations are VHL, BAP‐1, PBRM‐1, SETD2, MTOR, and KDM5C; along with this, the genetic modifications in 5q, 14q, 7q, 8p, and 9p are also responsible for RCC manifestation (Hsieh et al. 2017). The tumor suppressor gene PBRM‐1, which encodes the BAF180 protein, is a major contributor to RCC prevalence. Animal studies have stated cell cycle regulation and replicative senescence; thus, altered PBRM‐1 results in an abnormal BAF180, resulting in uncontrolled cell growth and tumorigenesis (Liu et al. 2020).

Chemical‐induced oxidative stress (OS) plays a significant role in gene mutations, thus contributing to cancer progression via altering physiological and biochemical mechanisms. Various studies have proved Naringenin's therapeutic and preventive role in chemical‐induced renal damage and oxidative stress. Alaqeel and Al‐Hariri (2023) reported the nephroprotective role of Naringenin via attenuating TNF‐α, IL6, COX‐2, NF‐ƘB, and KIM‐1 expression. They fed Wistar rats with 100 and 200 mg/kg body weight Naringenin to evaluate its potential and found that administration of Naringenin improved renal function and enhanced antioxidant capacity. Khaled et al. (2022) proved the antioxidant potential of Naringenin (10 mg/kg) by improving GSH, SOD, and GPx, while decreasing MDA levels, NO, PGE‐2, and lipid peroxidation in paclitaxel‐induced renal injury in Wistar rats. Naringenin (50 and 100 mg/kg) for 21 days reduced oxidative stress in doxorubicin‐induced nephrotoxic animals (Wistar rats) by lowering inflammatory markers such as NO, TNF‐α, IL‐6, IL‐1α, PGE‐2, and NOX4 (Khan et al. 2020). Wang, Xie, et al. (2021) described Naringenin's role in PTEN/PI3K/AKT pathway regulation in RCC. They stated that Naringenin (0, 1, 2, 4, 6, 8, 10, 12, 14 or 16 μm) induced cytotoxicity by diminishing Ki67 expression, downregulating Bcl‐2 and PI3K expression, and augmenting apoptosis via upregulating caspase‐8 and PTEN in 786‐O and OS‐RC‐2 cell lines. Moreover, Naringenin (0, 4, or 8 μm) reduced cell proliferation and initiated cell apoptosis.

### 2.12 Gastric Cancer

The stomach is the key organ associated with food digestion with the help of gastric secretions and acid. Furthermore, it also acts as a food reservoir and involves gastrointestinal motility. The stomach/gastric cancer is affecting 6 of every 10 people above 65, and the prevalence is higher among men (1 in 101) rather than females (1 in 155). According to the statistics of ACS, 26,890 new cases of stomach cancer and ~10,880 deaths from this type of cancer will be estimated in 2024. The main root cause of gastric cancer is dietary behavior along with lifestyle habits. The *H. pylori* infection, salty foods, obesity, smoking, alcohol, Epstein–Barr virus, nitroso compounds, low folate intake, and occupational exposures are major risk factors for gastric cancer (Thrift and El‐Serag 2020). Most previous studies have affirmed the direct association of *H. pylori* with gastric carcinoma, and a suitable environment with an impaired immune system of the host can increase the risk of cancer incidence. These gram‐negative bacteria secrete multiple substances like urease, acetaldehyde, protease, phospholipase, and ammonia, resulting in mucosal damage through urease‐mediated myosin II stimulation (Dincă et al. 2022). *H. pylori* is involved in the production of ROS and oxidative stress, leading to DNA damage via NF‐κB, and Wnt/β‐catenin activation is a significant factor in gastric carcinoma pathogenesis (Zhang et al. 2022).

Dietary behaviors such as excess salt and salty foods have been reported in gastric cancer development. Saturated NaCl (S‐NaCl) encourages the development of N‐methyl‐N′‐nitro‐N‐nitrosoguanidine‐induced gastric carcinomas (Balendra et al. 2023). The MSI, CIN, genetic mutations in oncogenes, and TSGs are crucial factors in gastric carcinoma. The alteration in K‐ras oncogene, overexpression of cell surface receptor c‐erbB2 of the tyrosine kinase family, and irregularities in the FGFR2/ErbB3/PI3 kinase pathway have been widely linked with gastric cancer (Zhu 2020). The dysfunction of TSG p53 characterized by GC‐AT transitions caused by carcinogenic N‐nitrosamines and *PTEN* inactivation on chromosome 10q23.31 are major contributors to carcinoma. Another TSG, RUNX3, is also tangled in the complex process of stomach oncogenesis (Song et al. 2020). Numerous growth factors are also involved in the progress of gastric carcinoma, and alterations in these growth factors further produce mediators that worsen the conditions. The increased expression of TGFBR2, CDC25A, SMAD7, and RELA and downregulation of p27 are major events in cell proliferation and cancer progression (Kumari et al. 2021).

Evidence‐based studies have proved Naringenin's therapeutic and anticancer potential to ameliorate gastric carcinoma. Recently, Liu et al. (2024) studied Naringenin‐loaded bovine serum albumin nanoparticles to prevent gastric cancer and ulcers. They administered rats with nanoparticles and found inhibited gastric carcinoma cell proliferation and migration, apoptosis induction, and reduced TNF‐*α* mRNA expression and ROS. Thus, these nanoparticles effectively postpone gastric cancer cell development. Advanced computational techniques like molecular docking and network pharmacology are promising approaches to estimating the therapeutic potential of bioactive compounds. In this context, Jingjie et al. (2021) investigated the preventive approach of Naringenin against gastric carcinoma based on a biomolecular network. They studied 61 targets like TP53, MAPK3, AKT1, Casp3, ESR1, ALB, and EGFR in Naringenin mechanisms and reported apoptosis initiation via PI3 K‐AKT, p53, and NF‐κB pathways regulation. Naringenin could prove more beneficial because of its natural and safe origin and ability to target multiple factors. In contrast, ABT‐263 only targets specific factors and has some adverse effects like nausea, vomiting, and reduced appetite. Ye LiQun et al. (2019) studied the combined impact of Naringenin and the Bcl‐2 inhibitor ABT‐263 on SGC7901 gastric cancer cell death and proliferation. The results proved that combined and separate treatments both proved effective in apoptosis promotion and cell proliferation inhibition at 48 h via regulating AKT pathways. Previously, Bao et al. (2016) and Zhang et al. (2016) also demonstrated the anticancer potential of Naringenin and Naringenin combined with ABT‐737 against SGC‐7901 gastric cancer cells in their separate studies. Both studies concluded that Naringenin (0, 5, 10, 20, 40, 80, 160 μmol/L) alone or combined can substantially inhibit proliferation in cancer cell lines through upregulating caspase3 and p53 expression and dysregulating AKT—moreover, 0, 20, 40, or 80 μmol/L of Naringenin induced apoptosis in cancer cells.

### 2.13 Bladder Cancer

Bladder cancer is common among males rather than females, affecting 1 in 28 men and 1 in 89 women over the age of 55 years or above. According to ACS, 4% of cancer cases are bladder cancers in the US, and 83,190 new cases of bladder cancer (63,070 men and 20,120 women) and 16,840 deaths from bladder cancer will be estimated in 2024. A family history of bladder cancer, genetic mutations, exposure to dyes and chemicals, UTI, and high arsenic content in drinking water are major risk factors for bladder cancer (Alouini 2024). Bladder cancer can be divided into two types because of its progression and aggressiveness to penetrating bladder walls. The first type is non‐muscle‐invasive bladder cancer, covering ~75% of cases of bladder cancer, and the second is muscle‐invasive bladder cancer, responsible for the rest of the 25% of cases (van Straten et al. 2023). The pathological studies have suggested that increased RONS, MDA, NO, 8‐iso‐PGF2α, and declined SOD2 levels play significant roles in bladder cancer progression. Moreover, a series of genes such as NAT2, TP63, GSTM1, MYC, TACC3‐FGFR3, PSCA, CLPTM1L‐TERT, APOBEC3A‐CBX6, UGT1A, and CCNE1 get mutated in bladder cancer. The overproduction of RONS causes NF‐κB activation, cytokine production, and NOS2 synthesis, leading to ROS/MAPK, ROS/Keap1‐Nrf2‐ARE, and ROS/PI3K/Akt pathways stimulation, linked with bladder cell proliferation (Grębowski et al. 2023).

The ROS and RNS are involved in the damage of nucleic acids and activate pro‐inflammatory cytokines like IL‐6 and IL‐8, which worsen the activity of biochemical pathways and promote malignancy. Thus, inflammatory cytokines and their receptors may enhance malignant transformation and bladder cancer progression by activating transcription factors. Therefore, mutations in any of these factors are linked to gene mutation and abnormal cell growth. Additionally, RBPs and miRNAs are critical in gene regulation and protein expression (Behzadi et al. 2022). Previous studies have proved that RBM3, an RBP, binds to the 60S ribosomal subunit and affects the mRNA stability of IL‐8, thus promoting eIF4E phosphorylation. However, RNA‐binding protein LIN28 regulates the IL‐6 expression and reduces let‐7 maturation, resulting in NF‐κB activation and neoplasm development (Gao et al. 2023). The research on the anticancer activity of Naringenin against bladder cancer has proved its effectiveness. Carvalho Radicchi et al. (2022) studied the anti‐tumor effect of naringin in silico and in vitro on bladder cancer cells and concluded that naringin (2.5–400 μM) showed cytotoxic effects, decreased the colony number, stopped cell migration, altered morphology and cell cycle development of evaluated two cell lines. Previously, Liao et al. (2014) proved that Naringenin (0–300 μM) could inhibit cell growth and cancer development against TSGH‐8301 bladder cancer cells via downregulating AKT and MMP‐2 pathways.

### 2.14 Uterine Cancer

Uterine cancer is a common cancer of the female reproductive organs, affecting women aged above 60. According to ACS, it has been estimated that 67,880 new cases of uterus cancer will be diagnosed, and 13,250 women will die from uterus/uterine cancers in 2024. Uterine cancer can be categorized as endometrial, which develops from the lining of the uterus, accounting for 95% of cases, and myometrial or uterine sarcomas, which grow in the muscle tissue and are a rare form of uterine cancer (David et al. 2020). Endometrial cancer (EC) is associated with several risk factors, such as obesity, certain medications, hormonal imbalance, PCOS, age, T2DM, family history, and previous history of breast or ovarian cancer. Genetic mutation is a fundamental and crucial aspect of EC development involving the modification of MMR genes, that is, MLH1, MSH2, PMS2, and MSH6. Moreover, the difference between the BRCA gene family and EC remains unclear. However, the links between BRCA1 and BRCA2 have been proven by some studies (Long et al. 2019). The pathways, such as SDF‐1alpha/CXCR4 and HGF/c‐Met, activate kinases like MAPK, PI3K/AKT, which phosphorylate ER and cause activation of CDKN1A and specific growth factors, that is, IGF‐1, TGF, and MAD2L1, leading to cell proliferation and malignancy (Winuthayanon et al. 2017). Several studies have also proven that uterine carcinosarcomas (UC) occur because of genetic alterations, and these genetic mutations are contributing to UC progression. The genes include ARID1A (10%–25%), PTEN (10%–50%), KRAS (10%–15%), TP53 (60%–90%), PPP2R1A (15%–30%), and FBXW (10%–40%) are reported for UC cases, and TP53 and FBXW7 mutations are most frequent in UC (Urick and Bell 2019; Cuevas et al. 2019).

The anticancer potential of Naringenin against EC cells was proved by the study of Aichinger et al. (2018), as they investigated the antiproliferative activity of Naringenin and xanthohumol against fusarium mycotoxins in Ishikawa EC cells. They concluded that both compounds (CI50 2.12) have a significant antagonist role against erogenicity in EC via inhibiting the AlP‐inducing effect and suppressing cell proliferation. However, xanthohumol may have a slight edge because of its unique ability to be converted into other bioactive compounds like 8‐prenylnaringenin. Endometriosis (EMS) is a gynecological problem caused by oxidative stress and ROS, considered precursor lesions of malignancies and endometriosis‐associated carcinoma, and may lead to endometrial hyperplasia. However, the direct association of EMS and EC is still unclear, but because of the potent risk factor, it cannot be neglected. Studies have evidenced that Naringenin has the potential to ameliorate EMS. Considering this, Kapoor et al. (2019) proved that Naringenin (50 mg/kg/day) for 21 days alleviated the development of endometriosis by regulating the Nrf2/Keap1/HO1 axis, inhibiting prognostic markers (VEGF, TAK1, PCNA, PAK1) expression, inducing apoptosis in female Sprague Dawley rats and decreasing MMP‐2 and MMP‐9 expression in in vitro culture. Park, Lim, et al. (2017) studied the Naringenin effect on EMS in VK2/E6E7 and End1/E6E7 endometriosis cell lines. They stated that Naringenin inhibited proliferation and amplified apoptosis via Bax and Bak apoptotic proteins, MAPK activation, and inactivation of PI3K pathways. Moreover, Naringenin improved ROS and ER stress by stimulating GADD153, eIF2α, IRE1α, and GRP78 proteins.

### 2.15 Bone Cancer

Bone cancer is a less common cancer, accounting for < 1% of all cancers, and it affects almost every age of people. According to ACS, 3970 new cases have been diagnosed (2270 males and 1700 females), and 2050 deaths (1100 males and 950 females) will be estimated from bone cancer in 2024. Osteosarcoma is the most common type of bone cancer, followed by chondrosarcoma and Ewing tumors/Ewing sarcoma (Hu et al. 2023). Several risk factors, including age, hormones, diet, alcohol, obesity, sunlight, pathogens, inflammation, chemicals, and toxins, contribute to bone cancer prevalence. Osteosarcoma is credited to chromosomal aberration and changes in p53, Rb1, and deoxyribonucleic acid repair genes, whereas chondrosarcoma is linked with EXT1/2, TP53, Rb1, and IDH1/2 gene alterations. The last Ewing sarcomas are due to chromosomal translocations, leading to the melding of an FET protein to an ETS transcription factor, most frequently FLI1 (Chow 2018; Grünewald et al. 2018).

Osteosarcoma, the most common bone cancer, has been frequently discussed in the literature. The studies have verified that various factors are involved in malignancy. The p53 and retinoblastoma (Rb) genes are renowned TSGs, and dysfunction of these genes is a crucial factor in cancer progression. 50% of all cancers and 22% of osteosarcomas are due to p53 gene mutation. The p53 gene shows its tumor‐suppressor effects through activating proapoptotic Bax and p21. The latter inactivates G1/S‐Cdk and S‐Cdk complexes, leading to cell cycle arrest in G1 (Pan et al. 2021). The transcription factor AP‐1 complex that regulates cell division, differentiation, and bone development is compromised by Fos and Jun proteins, part of the c‐fos and c‐jun proto‐oncogenes. Jun and Fos are significantly involved in high‐grade osteosarcomas and metastasis development (Lv et al. 2019). Overexpression of Myc, another transcription factor involved in cell division stimulation, has been reported in osteosarcoma, and downregulation of Myc results in cell cycle arrest. Similarly, growth factors like TGF, CTGF, IGF, and TGF‐β are crucial in bone matrix synthesis, proliferation, and apoptosis. Furthermore, bone morphogenic proteins (BMPs) are a major part of the TGF‐β family and are associated with high‐grade osteosarcomas (Xie et al. 2023).

Numerous studies have proved the therapeutic potential of Naringenin against osteosarcoma through autophagy, apoptosis, and cell cycle arrest. Recently, Li, Zheng, et al. (2024) elaborated on the role of Naringenin in bone cancer pain (BCP) through the NF‐κB/uPA/PAR2 pathway in C57BL/6 mice. They concluded that Naringenin (50 mg/kg/day) i.p. for 21 days recovered BCP damage and reduced PAR2, PKC‐γ, PKA, TRPV1, p‐IKKβ, p‐p65, and uPA levels in bone tissues. Lee et al. (2022) studied the antiproliferative activity of Naringenin against HOS and U2OS cell lines. They concluded that Naringenin (100, 250, and 500 μM) stopped cell growth and induced cell cycle arrest via downregulating B1 and cyclin‐dependent kinase 1 and upregulating p21 expression. Moreover, Naringenin upregulated ER stress markers, such as GRP78 and GRP94, and stimulated ER stress‐mediated apoptosis. Ge et al. (2022) proved that Naringenin (100 mg/kg, i.p.) reduced BCP in Sprague Dawley female rats via modulating the AMPK/PGC‐1α signaling axis. It also improved GPx4 levels, reduced neuroinflammation through inhibited NF‐κB p65 expression, and promoted microglial polarization. Likewise, Song and Liu (2021) showed that Naringenin alleviated CIBP in a xenograft model of Sprague–Dawley rats via injecting Walker 256 tumor cells. The rats were treated with low, medium, and high doses (16.7, 50, and 150 mg/kg/day) for 10 days. The findings showed that Naringenin reduced CIBP by modulating the P2X7R/PI3K/AKT pathway via reducing TNF‐α, IL‐1β, and IL‐6. Therefore, it can be a therapeutic strategy to manage bone cancer and its pain. Chang et al. (2020) reported that Naringenin (25, 50, 100, 200, and 300 mM) inhibited cell proliferation and migration in bone cancer cells (143B) by downregulating MMP‐2 and 9.

### 2.16 Cervical Cancer

Cervical cancer (CC) is the 4th common cancer among women, with 660,000 new cases and about 350,000 deaths reported in 2022. According to ACS estimation, 13,820 new cases of invasive CC will be diagnosed, and 4360 women will die from cervical cancer in 2024. The incidence rate is high among women aged 35–44 and low in younger females. Smoking, impaired immunity, multiple sex partners, and sexually transmitted pathogens are risk factors for cervical cancer. However, human papillomavirus (HPV) is known for more than 75% of CC cases (Zhang et al. 2020). Among various strains of HPV, types 16 and 18 are the most notorious because of causing high‐grade CC. The oncoproteins (E6, E7) of HPV disrupt the host cell cycle, especially E6, which disturbs the tumor suppressor protein p53, whereas E7 interacts with retinoblastoma protein (pRB). In addition, E6 can degrade other proteins involved in the apoptosis signaling cascade, like Bak, FADD, and procaspase 8. Alongside, the E5 protein may play a role in immune dysfunction and oxidative stress, and microRNAs also play a role in cervical carcinogenesis (Romero‐Masters et al. 2022).

Zhou et al. (2024) worked on molecular docking and network pharmacology of cervical cancer. Furthermore, an in vitro study was also conducted on HeLa cell viability and migration to highlight the potential of Naringenin in CC treatment. The outcomes showed that Naringenin (0 μM, 100 μM, and 200 μM) diminishes the migration of HeLa cells by downregulating the EGFR/PI3K/AKT signaling pathway. Martínez‐Rodríguez et al. (2021) explored Naringenin (500 μM) and cisplatin (16 μM) combined effect on CC cell toxicity, apoptosis, and proliferation in HeLa cell culture. They found the inactivation of caspases 3, 9, 7, and 8, indicating necrosis instead of apoptotic cell death and inactive cell proliferation in the G0/G1 phase. Lastly, Naringenin showed anticancer activity via molecular docking of RIP3 and MLKL, cyclin B, MMP 2, and MMP 9 proteins. Jia et al. (2021) studied the Naringenin‐cisplatin effect on HeLa/DDP cervical cancer cells through the AMPK pathway. They concluded that Naringenin‐cisplatin (60 + 2 mg/L) inhibited proliferation and activated apoptosis of the HeLa/DDP cell line by stimulating autophagy. Moreover, p‐AMPK/AMPK, Bax, Beclin1, and LC3 II protein expression were higher than LC3 I.

### 2.17 Limitations

Naringenin, a natural flavonoid with promising anticancer properties, faces several limitations that hinder its clinical application. Its poor water solubility and low oral bioavailability significantly reduce its systemic absorption and therapeutic efficacy. Rapid metabolism and elimination further limit its bioactive concentration in target tissues. The variability in individual metabolism also contributes to inconsistent outcomes. Moreover, limited clinical trials exist to confirm its safety and efficacy in humans. These challenges highlight the need for advanced drug delivery systems like nanoparticles to enhance their anticancer potential.

## 3 Conclusion and Future Perspective

Naringenin is a flavonoid bioactive compound mainly available in citrus fruits and recognized for antioxidant, anti‐inflammatory, and anticancer properties. The bioavailability and bioaccessibility of Naringenin can be affected by food sources, processing techniques, and gastrointestinal conditions. Naringenin is fermented by probiotics in the colon and synthesizing metabolites that improve immune function and reduce oxidative damage. Various in vitro and in vivo cancer studies have proved Naringenin's anticancer potential via modulating inflammatory and signaling pathways. Naringenin shows anticancer activity through apoptosis, necrosis, cell cycle arrest, and downregulation of oncogenes. However, future research on Naringenin should prioritize advanced in silico modeling to predict molecular target interactions and comprehensive, robust animal models to validate efficacy and safety. Improving stability via formulation innovations like nanoparticle encapsulation and prodrug approaches will enhance bioavailability. Phase I and II clinical trials, guided by comprehensive pharmacokinetic and toxicological data, are essential to translate preclinical successes into anticancer therapies, ultimately unlocking Naringenin's full therapeutic potential.

## Author Contributions

**Ahmad Mujtaba Noman:** conceptualization (equal), writing – original draft (equal). **Muhammad Tauseef Sultan:** conceptualization (equal), writing – original draft (equal). **Hassan Raza:** data curation (equal), writing – original draft (equal). **Muhammad Imran:** investigation (equal), writing – review and editing (equal). **Muzzamal Hussain:** investigation (equal), writing – original draft (equal). **Ahmed Mujtaba:** formal analysis (equal), validation (equal), visualization (equal). **Ehab M. Mostafa:** data curation (equal), methodology (equal). **Mohammed M. Ghoneim:** resources (equal), validation (equal), visualization (equal). **Samy Selim:** investigation (equal), software (equal), visualization (equal). **Soad K. Al Jaouni:** data curation (equal), investigation (equal). **Mohamed A. Abdelgawad:** investigation (equal), validation (equal), visualization (equal). **Tadesse Fenta Yehuala:** data curation (equal), investigation (equal), supervision (equal), writing – review and editing (equal). **Suliman A. Alsagaby:** data curation (equal), writing – review and editing (equal). **Waleed Al Abdulmonem:** investigation (equal), writing – review and editing (equal).

## Conflicts of Interest

The authors declare no conflicts of interest.

## Data Availability

The data that support the findings of this study are available on request from the corresponding author.
